# Genetic Variants of MicroRNA and DROSHA Genes in Association With the Risk of Tuberculosis in the Amazon Population

**DOI:** 10.3389/fgene.2022.850058

**Published:** 2022-03-02

**Authors:** Diana Feio da Veiga Borges Leal, Mayara Natália Santana da Silva, Lucas Favacho Pastana, Marianne Rodrigues Fernandes, Aidalucy do Socorro Costa de Athayde, Débora Christina Ricardo Fernandes Porchera, Cleonardo Augusto da Silva, Antônio André Conde Modesto, Paulo Pimentel De Assumpcão, Sidney Emanuel Batista dos Santos, Ney Pereira Carneiro dos Santos

**Affiliations:** ^1^ Núcleo de Pesquisa em Oncologia, Universidade Federal do Pará, Belém, Brazil; ^2^ Laboratório de Genética Humana e Médica, Instituto de Ciências Biológicas, Universidade Federal do Pará, Belém, Brazil

**Keywords:** genetic variants, microRNA, drosha, tuberculosis, risk factor

## Abstract

Tuberculosis (TB) is a chronic infection caused by *Mycobacterium tuberculosis* (Mtb) with high incidence and mortality. Studies reported that host genetic variants might be associated with the risk of tuberculosis. The aim of this study was to perform an association study between 26 single nucleotide polymorphisms (SNPs) and tuberculosis and evaluate whether these SNPs may confer risk factors to tuberculosis in the Amazon population. There were 52 males and 126 females, with total of 178 healthy controls. Genotyping was performed using TaqMan Open Array Genotyping. Ancestry-informative markers were used to estimate the ancestral proportions of the individuals in the case and control groups. The results indicated that the SNPs rs10035440 (DROSHA), rs7372209 (miR26-a1), rs1834306 (miR100), rs4919510 (miR608), and rs10739971 (pri-let-7a-1) were significantly associated with high risk and rs3746444 (miR499) and rs6505162 (miR423), with low risk of developing tuberculosis in the Amazon population. Our study concluded that seven miRNA polymorphisms were associated with tuberculosis. Our study contributes to a better understanding of TB pathogenesis and may promote the development of new diagnostic tools against *M. tuberculosis* infection.

## 1 Introduction

Tuberculosis (TB) is a chronic infection caused by *Mycobacterium tuberculosis* (Mtb), and it remains one of the most serious infections with high incidence and mortality, mostly in developing countries. According to the World Health Organization (WHO) report, there were an estimated number of 10 million new TB cases and 1.4 million deaths worldwide in 2019 (WHO 2020).

Early detection and appropriate treatment are the most important control strategy for TB. However, there is a low rate of TB cases detected and officially notified. In addition, smear microscopy and culture are the “gold standards” for the diagnosis of TB; however, faster and more sensitive diagnostic tests are required to improve diagnosis and patient management during infection (Acharya et al., 2020).

Emerging evidence suggests that miRNAs might be involved in the pathogenesis of a variety of human diseases, including TB (Sabir et al., 2018). MicroRNAs are an important regulator of biological processes, such as innate immune response against various infections (Wang and Cui 2012). They regulate the primary function of macrophages, dendritic cells, and natural killer cells (NKCs) and influence other regulation of proteins involved in the innate and adaptive immune pathways (Sahu et al., 2017; Tamgue et al., 2019).

Comparative miRNA expression profiles in individuals with active tuberculosis and healthy controls were studied in an Amazon population. Three different miRNA expressions, hsa-miR-4732-5p, hsa-miR-486-3p, and hsa-let-7g-5p, were identified in tuberculosis patients (Silva et al., 2021). However, different miRNA expressions can vary between individual samples during treatment and recovery (Kleinsteuber et al., 2013).

Recently, genetic variants in IL10, VDR, and microRNA genes were reported in subsequent studies in different human populations. However, there were few results of the association between these microRNA polymorphisms and tuberculosis susceptibility (Song et al., 2013; Silva et al., 2020). The aim of this study was to perform an association study between SNPs in miRNA and DROSHA and tuberculosis and evaluate whether these selected SNPs may confer susceptibility or protection to TB in the Amazon population.

## 2 Materials and Methods

### 2.1 Subjects

There were 52 males and 126 females, with total of 178 healthy controls in the Amazon population. The subjects enrolled in the case group were individuals diagnosed with active TB. The diagnosis of TB was based on clinical, radiological, sputum acid-fast bacillus (AFB) smear positivity, and/or culture. The patients were diagnosed with TB for a minimum of 6 months in Hospital João Barros Barreto in Belém city (Pará, Brazil). The control group was a special group with healthy individuals who were participants from a Geriatric Program in Hospital João Barros Barreto in Belém city (Pará, Brazil), with a high probability to be in contact with TB disease. The study was approved by the Ethical Committee of Universidade Federal do Pará, and all subjects gave written informed consent (protocol number: 42106720.2.0000.5634).

### 2.2 Single Nucleotide Polymorphism Selection

A total of 26 single nucleotide polymorphisms were selected based on previous reports of the marker in associative studies found in the Pubmed (www.ncbi.nlm.nih.gov/) database. Most SNPs are related to biological processes, immune responses, tuberculosis, or infectious diseases.

### 2.3 DNA Extraction

Peripheral blood (8 ml) from patients and healthy subjects was collected in EDTA-coated tubes. Genomic DNA from case and control groups was extracted from 200 uL peripheral whole blood samples using the Mini Spin Plus Extraction Kit (BioPur, Curitiba, Paraná, BR), according to the manufacturer’s instructions. The DNA samples were quantified by spectrophotometry at 260 nm using the Nanodrop 2000 device (NanoDrop Technologies, Wilmington, DE, United States). The purity of the analytes was evaluated through the ratio between absorbances obtained at the lengths of 260 and 280 nm, thus evaluating the quality of the DNA.

### 2.4 Single Nucleotide Polymorphism Genotyping

The polymorphisms were genotyped by allelic discrimination using TaqMan Open Array technology in the QuantStudio™12K Flex Real-Time PCR System (Thermo Fisher Scientific, Waltham, MA, United States), according to the manufacturer’s protocol. Data analysis was performed using the TaqMan^®^ Genotyper Software package (Thermo Fisher Scientific, Waltham, MA, United States).

### 2.5 Ancestry-Informative Markers

A panel of 61 ancestry-informative markers was used to estimate the ancestral proportions of an Amazon population described previously by Santos et al. (2010) and expanded later by Ramos et al. (2016). Brazil is a country with a highly admixed population, composed mainly of Europeans, Africans, and Native Americans. We used this AIM panel that was composed of ancestry-specific markers that can estimate individual and population genetic contribution.

### 2.6 Statistical Analysis

Differences in baseline characteristics, including age, sex, and ancestry analysis, were compared using Fisher’s exact and Mann–Whitney tests. The associations between SNPs and TB were estimated by computing the odds ratios (ORs) and 95% confidence intervals (95% CIs) using logistic regression analyses, adjusted for sex and ancestry. ORs were defined concerning the case groups, i.e., ORs >1 represent an increased risk of TB. A *p*-value < 0.05 was considered statistically significant. Hardy–Weinberg equilibrium (HWE) proportion tests with the Bonferroni correction were used to evaluate the quality of the genotype data.

## 3 Results

There were 52 males and 126 females, with total of 178 healthy controls (mean age 70.82 ± 1.8 years; 52 males and 126 females) were enrolled. Both significant differences were found in the distribution of age and sex between cases and healthy controls. The population structure results showed significant differences in European, Amerindian, and African contributions in the cases compared to controls. As expected, the population ancestry showed a higher Amerindian and African contribution and a lower European ancestry in the opposite direction in TB patients than that in controls (Table 1).

**TABLE 1:** Clinical characteristics and genetic ancestry of subjects.

Variable	Case (*n* = 190)	Control (*n* = 176)	*p*-value
Age (years)	51.8 (49.2–54.4)	70.82 (68.5–72.1)	<0.001^a^
Sex (male/female)	88(46.3%)/102(53.7%)	52(29.3%)/126(70.7%)	0.001^b^
EUR ancestry^b^	0.25 (0.23–0.27)	0.45 (0.42–0.48)	<0.001^c^
AMR ancestry^b^	0.42 (0.38–0.43)	0.31 (0.28–0.32)	<0.001^c^
AFR ancestry^b^	0.33 (0.31–0.35)	0.24 (0.22–0.26)	<0.001^c^

a Mann–Whitney test.

b Fisher’s exact Test.

c Multivariate regression logistic. Data are presented as median ±standard deviation.

A total of 26 SNPs were tested for the Hardy–Weinberg equilibrium with the Bonferroni correction (Table 2). In overall 26 SNPs, 10 markers were excluded for further analyses, and the remaining 16 SNPs were included for subsequent analysis by the Hardy–Weinberg equilibrium (Table 3). The results indicated that seven SNPs showed a significant association with TB risk, and those SNPs were rs10035440 in DROSHA, rs7372209 in miR26-a1, rs1834306 in miR100, rs4919510 in miR608, rs10739971 in pri-let-7a-1, rs3746444 in miR499, and rs6505162 in miR423 (Table 4).

**TABLE 2:** All 26 SNPs in microRNA and DROSHA genes with Hardy-Weinberg equilibrium (HWE) tests with the Bonferroni correction.

Gene	*p-value*
DROSHA (rs639174)	0.001
DROSHA (rs10035440)	0.787*
miR453 (rs56103835)	0.817*
miR 2053 (rs10505168)	0.109*
miR300 (rs12894467)	1*
miR-146A (rs2910164)	0.861*
miR196A2 (rs11614913)	1*
miR149 (rs2292832)	<0.001
AGO01 (rs636832)	0.850*
miR200B/200A/429 (rs9660710)	<0.001
miR20b/miR-17-5P (rs3660)	0.022
miR608 (rs4919510)	0.125*
miR499 (rs3746444)	0.204*
miR605 (rs2043556)	0.003
miR570 (rs4143815)	0.008
miR200C (rs12904)	0.340*
pre-miR938 (rs2505901)	<0.001
pri-let-7a-1 (rs10739971)	0.113*
miR219A1 (rs107822)	0.211*
miR604 (rs2368392)	0.002
miR26-a1 (rs7372209)	0.183*
miR100 (rs1834306)	0.718*
miR219-1 (rs213210)	<0.001
miR4513 (rs2168518)	0.073*
miR423 (rs6505162)	1*
DROSHA (rs3805500)	<0.001

**TABLE 3:** Association analysis of 16 SNPS with risk of TB disease in accordance with the Hardy–Weinberg equilibrium.

Genotype (SNP ID)	Case (%)	Control (%)	*p-value*	OR (95% CI)
DROSHA (rs10035440)	*n* = 107	*n* = 148	<0.001*	TT + CT vs. CC
CC	35 (33%)	06 (04%)		15.58 (5.04-48.15)^a^
CT	72 (67%)	44 (30%)	0.996	TT vs. CT + CC^b^
TT	0 (0%)	98 (66%)		
miR453 (rs56103835)	*n* = 75	*n* = 142	0.322	TT + CT vs. CC
CC	09 (12%)	07 (05%)		1.80 (0.56–5.77)^a^
CT	33 (44%)	54 (38%)	0.142	TT vs. CT + CC
TT	33 (44%)	81 (57%)		1.60 (0.85-3.00)^b^
miR 2053 (rs10505168)	*n* = 67	*n* = 122	0.975	TT + CT vs. CC
CC	10 (15%)	19 (15%)		1.05 (0.39-2.58)^a^
CT	29 (43%)	47 (39%)	0.931	TT vs. CT + CC
TT	28 (42%)	56 (46%)		1.03 (0.52–2.03)^b^
miR300 (rs12894467)	*n* = 146	*n* = 141	0.266	TT + CT vs. CC
CC	17 (11%)	20 (14%)		1.56 (0.71-3.44)^a^
CT	80 (55%)	46 (33%)	0.654	TT vs. CT + CC
TT	49 (34%)	75 (53%)		1.12 (0.66–1.90)^b^
miR146A (rs2910164)	*n* = 131	*n* = 160	0.696	GG + CG vs. CC
CC	13 (10%)	18 (11%)		0.845 (0.36-1.96)^a^
CG	59 (45%)	74 (46%)	0.590	GG vs. CG + CC
GG	59 (45%)	68 (43%)		1.15 (0.67–1.97)^b^
miR196A2 (rs11614913)	*n* = 96	*n* = 145	0.634	TT + CT vs. CC
CC	50 (52%)	65 (45%)		1.15 (0.64-2.07)^a^
CT	41 (43%)	65 (45%)	0.239	TT vs. CT + CC
TT	05 (05%)	15 (10%)		1.98 (0.63–6.18)^b^
AGO01 (rs636832)	*n* = 105	*n* = 150	0.643	GG + AG vs. AA
AA	15 (14%)	14 (09%)		1.22 (0.51-2.90)^a^
AG	45 (43%)	67 (45%)	0.450	GG vs. AG + AA
GG	45 (43%)	69 (46%)		0.80 (0.45–1.42)^b^
miR608 (rs4919510)	*n* = 116	*n* = 161	<0.001*	GG + CG vs. CC
CC	21 (18%)	85 (53%)		4.53 (2.30-8.92)^a^
CG	46 (40%)	58 (36%)	<0.001*	GG vs. CG + CC
GG	49 (42%)	18 (11%)		4.13 (2.25-7.60)^b^
miR499 (rs3746444)	*n* = 127	*n* = 167	0.995	GG + AG vs. AA^a^
AA	0 (0%)	126 (75%)		
AG	31 (25%)	36 (22%)	<0.001*	GG vs. AG + AA
GG	96 (75%)	05 (3%)		0.009 (0.003–0.26)^b^
miR200C (rs12904)	*n* = 106	*n* = 156	0.474	GG + AG vs. AA
AA	35 (33%)	38 (24%)		1.25 (0.67–2.33)^a^
AG	42 (39%)	72 (46%)	0.717	GG vs. AG + AA
GG	29 (28%)	46 (30%)		1.12 (0.60-2.07)^b^
pri-let-7a-1 (rs10739971)	*n* = 91	*n* = 83	<0.001*	GG + AG vs. AA
AA	70 (76.92%)	32 (38%)		6.14 (2.88-13.08)^a^
AG	17 (18.68%)	33 (40%)	<0.001*	GG vs. AG + AA
GG	4 (4.4%)	18 (22%)		7.25 (2.13-24.69)^b^
miR219-1(rs107822)	*n* = 90	*n* = 152	0.528	TT + CT vs. CC
CC	50 (55%)	85 (56%)		1.20 (0.67–2.17)^a^
CT	33 (37%)	53 (35%)	0.994	TT vs. CT + CC
TT	7 (8%)	14 (9%)		0.99 (0.34-2.87)^b^
miR26-a1 (rs7372209)	*n* = 113	*n* = 145	0.752	TT + CT vs. CC
CC	49 (44%)	70 (48%)		0.91 (0.52-1.59)^a^
CT	58 (51%)	56 (39%)	0.017*	TT vs. CT + CC
TT	6 (5%)	19 (13%)		3.75 (1.26–11.15)^b^
miR100 (rs1834306)	*n* = 76	*n* = 123	0.001*	GG + AG vs. AA
AA	35 (46%)	33 (27%)		3.39 (1.67–6.85)^a^
AG	31 (41%)	64 (52%)	0.128	GG vs. AG + AA
GG	10 (13%)	26 (21%)		1.99 (0.81-4.86)^b^
miR4513 (rs2168518)	*n* = 125	*n* = 163	0.505	GG + AG vs. AA
AA	9 (7%)	22 (13%)		0.73 (0.29–1.82)^a^
AG	51 (41)	61 (38%)	0.931	GG vs. AG + AA
GG	65 (52%)	80 (49%)		1.02 (0.60-1.73)^b^
miR423 (rs6505162)	*n* = 77	*n* = 124	0.021*	CC + AC vs. AA
AA	8 (10%)	30 (24%)		0.33 (0.13–0.84)^a^
AC	52 (68%)	62 (50%)	0.449	CC vs. AC + AA
CC	17 (22%)	32 (26%)		1.34 (0.62-2.90)^b^

*p*-values for logistic analyses of alternative models (dominant ^a^ and recessive ^b^ models) are shown. Multivariate logistic regression with sex and ancestry is used as covariates.

**TABLE 4:** Association analysis of seven significant SNPs with the risk of TB disease.

Genotype (SNP ID)	Case (%)	Control (%)	*p-value*	OR (95% CI)
DROSHA (rs10035440)	*n* = 107	*n* = 148	<0.001*	TT + CT vs. CC
CC	35 (33%)	06 (04%)		15.58 (5.04-48.15)^a^
CT	72 (67%)	44 (30%)	0.996	TT vs. CT + CC ^b^
TT	0 (0%)	98 (66%)		
miR608 (rs4919510)	*n* = 116	*n* = 161	<0.001*	GG + CG vs. CC
CC	21 (18%)	85 (53%)		4.53 (2.30-8.92)^a^
CG	46 (40%)	58 (36%)	<0.001*	GG vs. CG + CC
GG	49 (42%)	18 (11%)		4.13 (2.25-7.60)^b^
miR499 (rs3746444)	*n* = 127	*n* = 167	0.995	GG + AG vs. AA^a^
AA	0 (0%)	126 (75%)		
AG	31 (25%)	36 (22%)	<0.001*	GG vs. AG + AA
GG	96 (75%)	05 (3%)		0.009 (0.003–0.26)^b^
pri-let-7a-1 (rs10739971)	*n* = 91	*n* = 83	<0.001*	GG + AG vs. AA
AA	70 (76.92%)	32 (38%)		6.14 (2.88-13.08)^a^
AG	17 (18.68%)	33 (40%)	<0.001*	GG vs. AG + AA
GG	4 (4.4%)	18 (22%)		7.25 (2.13-24.69)^b^
miR26-a1 (rs7372209)	*n* = 113	*n* = 145	0.752	TT + CT vs. CC
CC	49 (44%)	70 (48%)		0.91 (0.52-1.59)^a^
CT	58 (51%)	56 (39%)	0.017*	TT vs. CT + CC
TT	6 (5%)	19 (13%)		3.75 (1.26–11.15)^b^
miR100 (rs1834306)	*n* = 76	*n* = 123	0.001*	GG + AG vs. AA
AA	35 (46%)	33 (27%)		3.39 (1.67–6.85)^a^
AG	31 (41%)	64 (52%)	0.128	GG vs. AG + AA
GG	10 (13%)	26 (21%)		1.99 (0.81-4.86)^b^
miR423 (rs6505162)	*n* = 77	*n* = 124	0.021*	CC + AC vs. AA
AA	8 (10%)	30 (24%)		0.33 (0.13–0.84)^a^
AC	52 (68%)	62 (50%)	0.449	CC vs. AC + AA
CC	17 (22%)	32 (26%)		1.34 (0.62-2.90)^b^

*p*-values for logistic analyses of alternative models (dominant ^a^ and recessive ^b^ models) are shown. Multivariate logistic regression with sex and ancestry is used as covariates. Genotypic frequency distributions are in accordance with the Hardy–Weinberg equilibrium (HWE, *p* > 0.05).

The results of the genotype distribution showed that the SNPs rs10035440 (DROSHA) and rs1834306 (miR100) were associated with an increased risk of TB (OR = 15.58, 95% CI [5.04-48.15] and OR = 3.39, 95% CI [1.67–6.85], respectively) in the dominant model. The SNP rs7372209 (miR26-a1) was associated with an increased risk of TB in a recessive model (OR = 3.75, 95% CI [1.26–11.15]).

In addition, the SNPs rs4919510 (miR608) and rs10739971 (pri-let-7a-1) were associated with an increased risk of TB in dominant and recessive models. The SNP rs4919510 was associated with an increased risk of TB in the analysis of the dominant model (OR = 4.53, 95% CI [2.30–8.92]) and in the analysis of the recessive model (OR = 4.13, 9 5% CI [2.25–7.60]). The SNP rs10739971 was associated with an increased risk of TB in the analysis of the dominant model (OR = 6.14, 95% CI [2.88–13.08]) and in the analysis of the recessive model (OR = 7.25, 95% CI [2.13–24.69]).

However, the SNPs rs3746444 and rs6505162 were associated with a low risk of TB in the analysis of the recessive model (OR = 0.009, 95% CI [0.003–0.26]) and rs6505162, in the analysis of the dominant model (OR = 0.33, 95% CI [0.13–0,84]).

## 4 Discussion

In the present study, we examined the possible association of polymorphisms in miRNA and DROSHA genes with tuberculosis susceptibility in the Brazilian Amazonian population. There were 52 males and 126 females, with total of 178 healthy controls. According to our results, we found seven SNPs in different genes that were associated with TB susceptibility. However, there are relatively few reports regarding the association of genetic variants and susceptibility to tuberculosis in an admixture of population such as the Brazilian Amazonian population.

Our previous study investigated the effect of ancestral background on TB susceptibility among the Brazilian Amazonian population. The results indicated that Amerindian (compared to African and European) ancestry populations were significantly associated as a risk factor for susceptibility to TB in the Brazilian Amazonian population (Leal et al., 2020). In the present study, we used ancestry-informative markers (AIMs) to estimate genetic ancestry and control for stratification to investigate genetic association among Brazilian admixed populations.

Our results suggested that the SNPs rs10035440 (DROSHA), rs4919510 (miR608), rs10739971 (pri-let-7a-1), rs7372209 (miR26-a1), and rs1834306 (miR100) were associated to an increased risk of TB. With respect to SNPs rs3746444 (miR499) and rs6505162 (miR423), these genetic variants were associated to a decreased risk for TB susceptibility in active-TB patients.

Previous studies have revealed that deregulation of miRNAs and miRNA-related genes occur in many diseases (de Souza et al., 2020; Silva et al., 2020). DROSHA initiates the biosynthesis of miRNAs by processing pri-miRNAs. Single nucleotide polymorphisms (SNPs) in miRNA biosynthesis genes, DROSHA, were indicated to be correlated with cancer risk as pediatric ALL (Gutierrez-Camino et al., 2014; López-López et al., 2014).

Some studies suggested that genetic variations were associated with cancer, HBV infection, and sepsis. Dai et al. (2018) reported that the rs4919510 polymorphism in miRNA-608 was associated with a decreased risk of collateral cancer (Dai et al., 2018). López-López et al. (2014) indicated significant associations between rs4919510 polymorphism in miRNA-608 and toxicity for cancer treatment (López-López et al., 2014). Al-Qahtani et al. (2017) found that miR-608 rs4919510 was associated with an increased risk of disease progression in patients with HBV (Al-Qahtani et al., 2017). Zhang et al. (2015) indicated that miR-608 rs4919510 was found to be strongly associated with a higher risk of developing sepsis and multiple organ dysfunction (Zhang et al., 2015).

miRNA let-7 is involved in numerous cellular processes, inflammation, immunity, protective functions, and viral infections (Bernstein et al., 2021). Li et al. (2016) reported the relationship of the pri-let-7a-1 rs10739971 polymorphism with the prognosis of gastric cancer patients in northern China (Li et al., 2016). *M. tuberculosis* suppresses the innate immune response in macrophages, when suppressing miRNA let-7f expression, and modulates the immune response to TB infection by elevating target expression, A20, an inhibitor of the NF-κB pathway (Kumar et al., 2015).

Ni et al. (2014) and Kleinsteuber et al. (2013) reported that *M. tuberculosis* can modulate the expression of host miRNA and limit the response of macrophages during TB infection. miRNA 26-A targets a component (P300) of the IFN-y signaling cascade and was found to be differently expressed during tuberculosis infection (Kleinsteuber et al., 2013; Ni et al., 2014). miRNA-26a regulates innate immune signaling, macrophages polarization, and trafficking of *Mycobacterium tuberculosis* to lysosomes during TB infection (Sahu et al., 2017). It also plays a role in intestinal inflammation (Zhang et al., 2021), preeclampsia (Eskandari et al., 2019), and cancer (Ying et al., 2016).

Studies have shown that miRNA-100 is associated with the adaptive immune system (Negi et al., 2015), anti-inflammatory function in the vascular system (Pankratz et al., 2018), and the occurrence, development, and cancer invasion (Liu et al., 2012; Huang et al., 2020). Genetic variation in miRNA-100 rs1834306 was found to be an increased risk of gastrointestinal disease (Zhu et al., 2020), increased risk of Hepatitis B virus (Motawi et al., 2019, 1834306), and a decreased risk for esophageal squamous cell carcinoma (Zhu et al., 2015) in several studies.

Li et al. (2011) reported that there was no association between miR-499 rs3746444 and pulmonary tuberculosis (PTB) risk in the Han population; however, subjects carrying the C allele showed a decreased PTB risk. Li et al. also found that there was an association between rs3746444 and PTB in the Tibetan population, and individuals carrying the C allele exhibited an increased PTB risk (Li et al., 2011). Naderi et al. (2015) indicated a lack of association between miRNA-146a rs2910164 and miRNA-499 rs3746444 gene polymorphisms and susceptibility to pulmonary tuberculosis (Naderi et al., 2015). Additionally, miR-499 rs3746444 was related to other diseases, including acute lymphoblastic leukemia (de Souza et al., 2020), asthma (Dong et al., 2020, 49), and inflammatory arthritis (Wang and Pan 2019).

Studies have evaluated the association between the SNP miRNA-423 rs6505162 and susceptibility to colorectal carcinoma (Jia et al., 2018) and coronary artery disease (Jha et al., 2019). A recent study showed that the expression of miRNA-423 was significantly increased in the serum of patients with tuberculosis, and the upregulation of miR-423-5p could suppress autophagosome–lysosome fusion in macrophages by posttranscriptional regulation of VPS33A (Tu et al., 2019).

## 5 Conclusion

In this study, it was possible to determine the genotypic frequencies for the SNP in selected genes as well as associations related to risk or protection for tuberculosis infection. We found seven genetic variants significantly associated in individuals with tuberculosis from the Amazon region. Our study revealed that the SNPs rs10035440 (DROSHA), rs7372209 (miR26-a1), rs1834306 (miR100), rs4919510 (miR608), and rs10739971 (pri-let-7a-1) were significantly associated with a high risk of TB. In addition, the SNPs rs3746444 (miR499) and rs6505162 (miR423) were significantly associated with a low risk of developing tuberculosis. Our study contributes to a better understanding of TB pathogenesis and may promote the development of new diagnostic tools against *M. tuberculosis* infection. Future studies must be carried out to evaluate the functional effects of polymorphisms of the selected genes as well as the inclusion of other SNPs and large sample study, to corroborate the findings for tuberculosis in our country.

## Data Availability

The datasets presented in this study can be found in online repositories. The names of the repository/repositories and accession number(s) can be found below: https://figshare.com/, https://doi.org/10.6084/m9.figshare.17954717.v2.

## References

[B1] Acharya B. Acharya A. Gautam S. Ghimire S. P. Mishra G. Parajuli N. (2020). Advances in Diagnosis of Tuberculosis: An Update into Molecular Diagnosis of *Mycobacterium T* . Mol. Biol. Rep. 47, 4065–4075. 10.1007/s11033-020-05413-7 32248381

[B2] Al-Qahtani A. A. Al-Anazi M. R. Nazir N. Wani K. Abdo A. A. Sanai F. M. (2017). Association of Single Nucleotide Polymorphisms in microRNAs with Susceptibility to Hepatitis B Virus Infection and HBV-Related Liver Complications: A Study in a Saudi Arabian Population. J. Viral Hepat. 24, 1132–1142. 10.1111/jvh.12749 28685993

[B3] Bernstein D. L. Jiang X. Rom S. (2021). let-7 microRNAs: Their Role in Cerebral and Cardiovascular Diseases, Inflammation, Cancer, and Their Regulation. Biomedicines 9, 606. 10.3390/biomedicines9060606 34073513 PMC8227213

[B4] Dai Z.-M. Lv J.-R. Liu K. Lei X.-M. Li W. Wu G. (2018). The Role of microRNA-608 Polymorphism on the Susceptibility and Survival of Cancer: a Meta-Analysis. Aging 10, 1402–1414. 10.18632/aging.101476 29909406 PMC6046227

[B5] de Souza T. P. de Carvalho D. C. Wanderley A. V. Fernandes S. M. Rodrigues J. C. G. Cohen-Paes A. (2020). Influence of Variants of the Drosha, Mir499a, and Mir938 Genes on Susceptibility to Acute Lymphoblastic Leukemia in an Admixed Population from the Brazilian Amazon. Am. J. Transl Res. 12, 8216–8224. 33437394 PMC7791525

[B6] Dong J. Sun D. Lu F. (2020). Association of Two Polymorphisms of miRNA-146a Rs2910164 (G > C) and miRNA-499 Rs3746444 (T > C) with Asthma: A Meta-Analysis. J. Asthma 58, 995–1002. 10.1080/02770903.2020.1759085 32308092

[B7] Eskandari F. Rezaei M. Mohammadpour-Gharehbagh A. Teimoori B. Yaghmaei M. Narooei-Nejad M. (2019). The Association of Pri-miRNA- 26a1 Rs7372209 Polymorphism and Preeclampsia Susceptibility. Clin. Exp. Hypertens. 41, 268–273. 10.1080/10641963.2018.1469643 29750583

[B8] Gutierrez-Camino A. Lopez-Lopez E. Martin-Guerrero I. Piñan M. A. Garcia-Miguel P. Sanchez-Toledo J. (2014). Noncoding RNA-Related Polymorphisms in Pediatric Acute Lymphoblastic Leukemia Susceptibility. Pediatr. Res. 75, 767–773. 10.1038/pr.2014.43 24618566

[B9] Huang C. Qin X. Zhao N. Jin H. Zhang S. Yang H. (2020). MicroRNA-100 Functions as a Tumor Suppressor in Cervical Cancer via Downregulating the SATB1 Expression and Regulating AKT/mTOR Signaling Pathway and Epithelial-to-mesenchymal Transition. Oncol. Lett. 20, 1336–1344. 10.3892/ol.2020.11686 32724376 PMC7377180

[B10] Jha C. K. Mir R. Elfaki I. Khullar N. Rehman S. Javid J. (2019). Potential Impact of MicroRNA-423 Gene Variability in Coronary Artery Disease. EMIDDT 19, 67–74. 10.2174/1871530318666181005095724 30289085

[B11] Jia W. Zeng L. Luo S. Bai F. Zhong R. Wu L. (2018). Association of microRNA-423 Rs6505162 C>A Polymorphism with Susceptibility and Metastasis of Colorectal Carcinoma. Medicine 97, e9846. 10.1097/MD.0000000000009846 29419695 PMC5944659

[B12] Kleinsteuber K. Heesch K. Schattling S. Kohns M. Sander-Jülch C. Walzl G. (2013). Decreased Expression of miR-21, miR-26a, miR-29a, and miR-142-3p in CD4⁺ T Cells and Peripheral Blood from Tuberculosis Patients. PLoS ONE 8, e61609. 10.1371/journal.pone.0061609 23613882 PMC3628900

[B13] Kumar M. Sahu S. K. Kumar R. Subuddhi A. Maji R. K. Jana K. (2015). MicroRNA Let-7 Modulates the Immune Response to *Mycobacterium T* Infection via Control of A20, an Inhibitor of the NF-Κb Pathway. Cell Host Microbe 17, 345–356. 10.1016/j.chom.2015.01.007 25683052

[B14] Leal D. F. D. V. B. Santana da Silva M. N. Fernandes D. C. R. D. O. Rodrigues J. C. G. Barros M. C. D. C. Pinto P. D. D. C. (2020). Amerindian Genetic Ancestry as a Risk Factor for Tuberculosis in an Amazonian Population. PLoS One 15, e0236033. 10.1371/journal.pone.0236033 32673332 PMC7365596

[B15] Li D. Wang T. Song X. Qucuo M. Yang B. Zhang J. (2011). Genetic Study of Two Single Nucleotide Polymorphisms within Corresponding microRNAs and Susceptibility to Tuberculosis in a Chinese Tibetan and Han Population. Hum. Immunol. 72, 598–602. 10.1016/j.humimm.2011.03.004 21524676

[B16] Li Y. Xu Q. Liu J. He C. Yuan Q. Xing C. (2016). Pri-let-7a-1 Rs10739971 Polymorphism Is Associated with Gastric Cancer Prognosis and Might Affect Mature Let-7a Expression. Onco Targets Ther. 9, 3951–3962. 10.2147/OTT.S100481 27445488 PMC4938131

[B17] Liu J. Lu K.-H. Liu Z.-L. Sun M. De W. Wang Z.-X. (2012). MicroRNA-100 Is a Potential Molecular Marker of Non-Small Cell Lung Cancer and Functions as a Tumor Suppressor by Targeting Polo-Like Kinase 1. BMC Cancer 12, 519. 10.1186/1471-2407-12-519 23151088 PMC3521172

[B18] López-López E. Gutiérrez-Camino Á. Piñán M. Á. Sánchez-Toledo J. Uriz J. J. Ballesteros J. (2014). Pharmacogenetics of MicroRNAs and MicroRNAs Biogenesis Machinery in Pediatric Acute Lymphoblastic Leukemia. PLoS One 9 (3), e91261. 10.1371/journal.pone.0091261 24614921 PMC3948785

[B19] Motawi T. K. Mady A. E. Shaheen S. Elshenawy S. Z. Talaat R. M. Rizk S. M. (2019). Genetic Variation in microRNA-100 (miR-100) Rs1834306 T/C Associated with Hepatitis B Virus (HBV) Infection: Correlation with Expression Level. Infect. Genet. Evol. 73, 444–449. 10.1016/j.meegid.2019.06.009 31176032

[B20] Naderi M. Hashemi M. Khorgami P. Koshki M. Ebrahimi M. Amininia S. (2015). Lack of Association between miRNA-146a Rs2910164 and miRNA-499 Rs3746444 Gene Polymorphisms and Susceptibility to Pulmonary Tuberculosis. Int. J. Mol. Cel Med 4, 40–45. PMC4359704 25815281

[B21] Negi V. Paul D. Das S. Bajpai P. Singh S. Mukhopadhyay A. (2015). Altered Expression and Editing of miRNA-100 Regulates iTreg Differentiation. Nucleic Acids Res. 43, 8057–8065. 10.1093/nar/gkv752 26209130 PMC4652766

[B22] Ni B. Rajaram M. V. S. Lafuse W. P. Landes M. B. Schlesinger L. S. (2014). *Mycobacterium T* Decreases Human Macrophage IFN-γ Responsiveness through miR-132 and miR-26a. J. Immunol. 193, 4537–4547. 10.4049/jimmunol.1400124 25252958

[B23] Pankratz F. Hohnloser C. Bemtgen X. Jaenich C. Kreuzaler S. Hoefer I. (2018). MicroRNA-100 Suppresses Chronic Vascular Inflammation by Stimulation of Endothelial Autophagy. Circ. Res. 122, 417–432. 10.1161/CIRCRESAHA.117.311428 29208678

[B24] Ramos B. R. D. A. D’Elia M. P. B. Amador M. A. T. Santos N. P. C. Santos S. E. B. da Cruz Castelli E. (2016). Neither self-Reported Ethnicity Nor Declared Family Origin Are Reliable Indicators of Genomic Ancestry. Genetica 144, 259–265. 10.1007/s10709-016-9894-1 26984822

[B25] Sabir N. Hussain T. Shah S. Z. A. Peramo A. Zhao D. Zhou X. (2018). miRNAs in Tuberculosis: New Avenues for Diagnosis and Host-Directed Therapy. Front. Microbiol. 9, 602. 10.3389/fmicb.2018.00602 29651283 PMC5885483

[B26] Sahu S. K. Kumar M. Chakraborty S. Banerjee S. K. Kumar R. Gupta P. (2017). MicroRNA 26a (miR-26a)/KLF4 and CREB-C/EBPβ Regulate Innate Immune Signaling, the Polarization of Macrophages and the Trafficking of *Mycobacterium T* to Lysosomes during Infection. Plos Pathog. 13, e1006410. 10.1371/journal.ppat.1006410 28558034 PMC5466338

[B27] Santos N. P. C. Ribeiro-Rodrigues E. M. Ribeiro-dos-Santos Â. K. C. Pereira R. Gusmão L. Amorim A. (2010). Assessing Individual Interethnic Admixture and Population Substructure Using a 48-Insertion-Deletion (INSEL) Ancestry-Informative Marker (AIM) Panel. Hum. Mutat. 31, 184–190. 10.1002/humu.21159 19953531

[B28] Silva C. A. Fernandes D. C. R. O. Braga A. C. O. Cavalcante G. C. Sortica V. A. Hutz M. H. (2020). Investigation of Genetic Susceptibility to *Mycobacterium T* (VDR and IL10 Genes) in a Population with a High Level of Substructure in the Brazilian Amazon Region. Int. J. Infect. Dis. 98, 447–453. 10.1016/j.ijid.2020.06.090 32619758

[B29] Silva C. A. Ribeiro-dos-Santos A. Gonçalves W. G. Pinto P. Pantoja R. P. Vinasco-Sandoval T. (2021). Can miRNA Indicate Risk of Illness after Continuous Exposure to *M. T*? Int. J. Mol. Sci. 22, 3674. 10.3390/ijms22073674 33916069 PMC8036329

[B30] Song X. Li S. QuCuo M. Zhou Y. Hu X. Zhou J. (2013). Association between SNPs in microRNA-Machinery Genes and Tuberculosis Susceptibility in Chinese Tibetan Population. Mol. Biol. Rep. 40, 6027–6033. 10.1007/s11033-013-2712-2 24078091

[B31] Tamgue O. Gcanga L. Ozturk M. Whitehead L. Pillay S. Jacobs R. (2019). Differential Targeting of C-Maf, Bach-1, and Elmo-1 by microRNA-143 and microRNA-365 Promotes the Intracellular Growth of *Mycobacterium T* in Alternatively IL-4/IL-13 Activated Macrophages. Front. Immunol. 10, 421. 10.3389/fimmu.2019.00421 30941122 PMC6433885

[B32] Tu H. Yang S. Jiang T. Wei L. Shi L. Liu C. (2019). Elevated Pulmonary Tuberculosis Biomarker miR-423-5p Plays Critical Role in the Occurrence of Active TB by Inhibiting Autophagosome-Lysosome Fusion. Emerging Microbes Infections 8, 448–460. 10.1080/22221751.2019.1590129 30898038 PMC6455132

[B33] Wang D. Pan G. (2019). Association of Rs2910164 Polymorphism in miRNA-146 and Rs3746444 Polymorphism in miRNA-499 with Inflammatory Arthritis: A Meta-Analysis. Biomed. Res. Int. 2019, 7305750. 10.1155/2019/7305750 31223622 PMC6541972

[B34] Wang J. Cui Q. (2012). Specific Roles of MicroRNAs in Their Interactions with Environmental Factors. J. Nucleic Acids 2012, 978384. 10.1155/2012/978384 23209884 PMC3502025

[B35] WHO (2020). Global Tuberculosis Report 2020. Global tuberculosis report 2020 Genebra.

[B36] Ying H.-Q. Peng H.-X. He B.-S. Pan Y.-Q. Wang F. Sun H.-L. (2016). MiR-608, Pre-miR-124-1 and Pre-miR26a-1 Polymorphisms Modify Susceptibility and Recurrence-free Survival in Surgically Resected CRC Individuals. Oncotarget 7, 75865–75873. 10.18632/oncotarget.12422 27713147 PMC5342784

[B37] Zhang A.-Q. Gu W. Zeng L. Zhang L.-Y. Du D.-Y. Zhang M. (2015). Genetic Variants of microRNA Sequences and Susceptibility to Sepsis in Patients with Major Blunt Trauma. Ann. Surg. 261, 189–196. 10.1097/SLA.0000000000000687 24743625

[B38] Zhang W. Fu X. Xie J. Pan H. Han W. Huang W. (2021). miR-26a Attenuates Colitis and Colitis-Associated Cancer by Targeting the Multiple Intestinal Inflammatory Pathways. Mol. Ther. - Nucleic Acids 24, 264–273. 10.1016/j.omtn.2021.02.029 33815939 PMC7985669

[B39] Zhu J. Yang L. You W. Cui X. Chen Y. Hu J. (2015). Genetic Variation in miR-100 Rs1834306 Is Associated with Decreased Risk for Esophageal Squamous Cell Carcinoma in Kazakh Patients in Northwest China. Int. J. Clin. Exp. Pathol. 8, 7332–7340. 26261633 PMC4525967

[B40] Zhu Y. Lin A. Zheng Y. Xie X. He Q. Zhong W. (2020). miR-100 Rs1834306 A>G Increases the Risk of Hirschsprung Disease in Southern Chinese Children. PGPM 13, 283–288. 10.2147/PGPM.S265730 32848443 PMC7428404

